# Immunogenicity and protective efficacy of seven candidate vaccines boosted with recombinant proteins, whole-cell bacterins of three serotypes of *Mannheimia haemolytica,* and an emulsion-type adjuvant

**DOI:** 10.3389/fvets.2025.1553396

**Published:** 2025-03-28

**Authors:** Aslı Balevi, Emine Eda Toslak, Ali Uslu, Zafer Sayın, Osman Erganis

**Affiliations:** Department of Microbiology, Faculty of Veterinary Medicine, Selcuk University, Konya, Türkiye

**Keywords:** antibody response, leukotoxin, *Mannheimia haemolytica*, S1-specific antigen, serotype 6, vaccines, challenge studies

## Abstract

**Introduction:**

*Mannheimia haemolytica* is a primary cause of bovine respiratory disease, leading to substantial economic losses in the livestock industry. Current commercial vaccines offer limited cross-serotype protection, and the rising prevalence of serotype 6 (S6) necessitates the development of more effective vaccines. This study aimed to develop novel candidate vaccines, including monovalent, bivalent, trivalent, and recombinant protein-based on S1, S2, and S6 serotypes of *M. haemolytica* formulations, to create an in-house ELISA with eight coating antigens.

**Methods:**

Five hundred lung samples from calves and sheep with respiratory infection symptoms were analyzed. Three *M. haemolytica* master seed strains (S1, S2, and S6) with diverse phenotypic and genotypic characteristics were selected. Recombinant leukotoxin (*lkt*) and S1-specific antigen (SSA-1) proteins were produced and used in the development of both vaccines and in-house ELISA. The eight coating antigens utilized were derived from whole-cell pellets, supernatant proteins of S1, S2, and S6, and recombinant *lkt* and SSA-1. Seven candidate vaccines (three monovalent, one bivalent, one trivalent, and two recombinant) were formulated with Montanide™ ISA 206 VG or Freund’s complete adjuvant. Female Swiss albino mice (*n* = 18 per group) were vaccinated twice at 21-day intervals via the intramuscular route.

**Results:**

S6 strains had the highest prevalence, with 43.07%. Interestingly, S6 strains expressed a prominent band at approximately 250 kDa, potentially causing haemorrhagic effects in mice. The S2 pellet performed best as an ELISA-coating antigen. The trivalent vaccine with Montanide™ ISA 206 VG provided the best protection in mice. Seropotency vaccine efficacy and challenge vaccine efficacy of trivalent vaccine were 95.8 and 100%, respectively. According to multinomial logistic regression analysis, the greatest odds ratio (0.97) was obtained from the trivalent vaccine.

**Conclusion:**

The haemorrhagic effects observed with S6 highlight the importance of including this serotype in future vaccines. The trivalent S6 vaccine with Montanide™ ISA 206 shows promise for improved protection against diverse *M. haemolytica* strains. Further research, including challenge studies in target animals, is needed to confirm these findings and evaluate field efficacy.

## Introduction

The bovine respiratory disease complex (BRDC) is one of the most significant health concerns in the cattle industry, causing substantial economic losses globally (1, 2). *M. haemolytica*, a primary bacterial agent associated with BRDC, contributes to bronchopneumonia in farm animals (3). *M. haemolytica* is a leading cause of morbidity and mortality in both dairy and beef cattle (2, 4). Cross-protection across serotypes has been an impediment to achieving effective BRDC control (5). The most prevalent serotypes of *M. haemolytica* are Serotype (S)1, S2, and S6, with an increasing prevalence of S6 observed in recent years (5–7). Current commercial vaccines offer limited cross-serotype protection, and the rising prevalence of S6 strains necessitates the development of more effective vaccines to address this issue (5). Leukotoxin (*lkt*) and outer membrane protein A (ompA) have been identified as promising vaccine candidates for *M. haemolytica* (2, 5, 7). *M. haemolytica* capsular serovars are not yet well characterized in relation to virulence and vaccine efficacy (2). An overall dissimilarity of 12% among *lkt* genes among various serovars suggests a need to explore strain variation and develop multivalent vaccines (2, 7). The S1-specific antigen (SSA-1), is the most conserved across *M. haemolytica* strains (6, 7). SSA-1 vaccine has exhibited strong immunogenicity and protection against challenge with heterologous strains in sheep, but its efficacy in cattle remains unknown (3, 5–7). Montanide™ ISA 206 VG has been used in veterinary vaccines for foot-and-mouth disease; it has demonstrated enhanced immunogenicity and protection compared to traditional alum adjuvant (with or without saponin) (8). Control of pneumonic pasteurellosis with monovalent commercial vaccines is time-consuming and has limited protection. Therefore, multivalent vaccines combined with adjuvants that can stimulate the immune response effectively and for a long time offer a good solution with good immunity.

**Figure 1:**
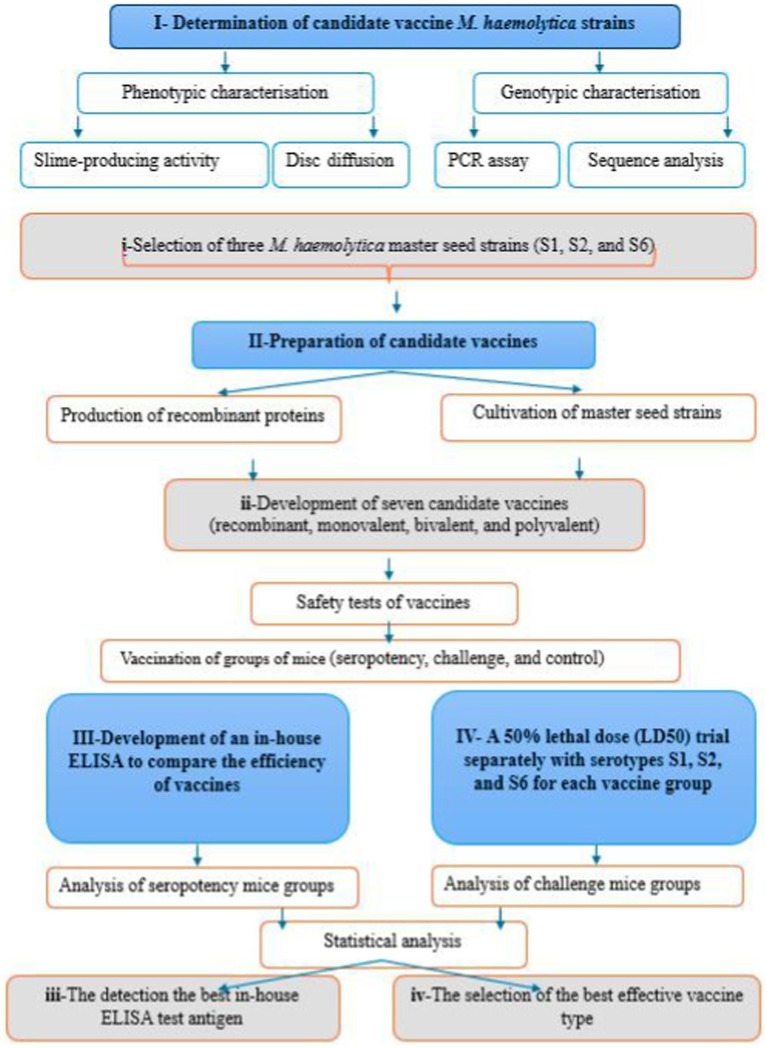
Timeline flowchart of the experimental design.

This study aimed to investigate the phenotypic and genotypic characteristics of S1, S2, and S6 strains, to select the vaccine strains (master seeds) for developing seven candidate vaccines (monovalent, bivalent, and trivalent), and to compare the efficiency of vaccines by an in-house made ELISA, which used eight different coating ELISA antigen. The working flowchart of *M. haemolytica* vaccines preparation is shown in Figure 1.

## Methods

### Ethics approval

Infected samples analysis was approved by the Faculty of Veterinary Medicine Ethics Committee at the University of Selcuk in Konya, Türkiye [grant number: 2020–69]. Murine model experiments were approved by the Experimental Medicine Research and Application Center Ethics Committee of Selcuk University in Konya, Türkiye [grant number:2019/38].

### Identification of *Mannheimia haemolytica* strains from ruminant

Lung samples (*n* = 500) from calves and sheep with at least one of the clinical symptoms of respiratory infection were used in this study. These samples were sent from distinct farms located in Turkey to the Faculty of Veterinary Medicine, Microbiology Laboratory for diagnosis from 2008 to 2018. A small piece of lung tissue (1.5 × 1.5 cm) was minced with sterile scissors and inoculated into a 4-ml volume of brain heart broth (110493, Merck). It was incubated at 37°C in 7% CO_2_ for 24 and 48 h, and then 100 μL of this preculture was spread on brain heart agar medium (103870, Merck) supplemented with 5% sheep blood, and it was incubated under the same conditions. Suspected *M. haemolytica* isolates were confirmed using a two-step procedure: standard biochemical procedures (Indole-production, Methyl red, Voges-proskauer, Simmons Citrate, Kliglerʼs Iron Agar, Urease, Motility, Maltose, Sucrose, Mannitol, Glucose, Cytochrome oxidase, Catalase) (6, 9) and Polymerase chain reaction (PCR) (10). Also, type strains (*M. haemolytica* ATCC 43270, *M. haemolytica* ATCC 29694, and *M. haemolytica* ATCC 29697) were used as positive controls in this study.

### Characterization of slime the producing ability of *Mannheimia haemolytica*

All strains were cultured on Congo red agar (CRA), prepared by adding 0.8 g of Congo red (C6767, Sigma Aldrich) and 36 g of saccharose to 1 L brain heart infusion (BHI) agar (103,870, Merck). The plates were subsequently incubated for 24 h at 37°C. Slime-producing strains developed black colonies in contrast to non-producing strains (11). Biofilm production in bacteria was one of the positive criteria in the selection of vaccine strains.

### Antibiotic resistance of *Mannheimia haemolytica* by disk diffusion test

Antibiotic resistance of the strains was determined on Mueller Hinton agar (1.05437, Merck) with 5% sheep blood as per the Clinical and Laboratory Standards Institute (CLSI). *Escherichia coli* ATCC 25922 was used as a control organism. Antimicrobial agents [amoxycillin-clavulanic acid (30 μg) susceptible<19 mm ≤ resistant (CT0223B, Thermo Scientific); ceftriaxone (30 μg) susceptible≤22 mm ≤ intermediate≤25 mm ≤ resistant (CT0417B, Thermo Scientific), gentamicin (10 μg) susceptible≤14 mm ≤ intermediate ≤17 mm ≤ resistant (CT0024B, Thermo Scientific), cefotaxime (30 μg) susceptible≤17 mm ≤ intermediate ≤20 mm ≤ resistant (CT0166B, Thermo Scientific), ceftazidime (30 μg) susceptible≤19 mm ≤ intermediate≤22 mm ≤ resistant (CT0412B, Thermo Scientific), ampicillin (10 μg) susceptible≤14 mm ≤ intermediate≤22 mm ≤ resistant (X90067, Oxoid), and ciprofloxacin (5 μg) susceptible≤22 mm ≤ intermediate≤25 mm ≤ resistant (X90076, Oxoid)] were added to the agar and incubated aerobically for 24 h. Results were evaluated by calculating the diameter of inhibition zones (in mm) (12). Depending on whether the isolates developed resistance to at least one or two antimicrobial groups, criteria of extensively drug resistance (XDR) or multi-drug resistance (MDR) were used. Multidrug resistance development in bacteria was one of the positive criteria in the selection of vaccine strains.

### Genotypic characteristics of *Mannheimia haemolytica* strains by PCR

DNA extracts were obtained using a Wizard™ Genomic DNA Purification Kit (A1120, Promega). The detection of 16S rDNA (10), serogroup (13), *lkt* (14), SSA-1 (15) was performed according to previously described protocols (Table 1). To investigate the presence of resistance genes against the following antimicrobial agents after these bacteria, genes (tetracycline (tetH)) (16), ampicillin (blaROB-1) (16), a quinolone (gyrA, parC) (17), tilmicosin (ermX) (18), and neomycin (aphA-1) (19) were analyzed by PCR (Table 1). Alternate serogroup 6 primers were designed using the NCBI program^1^ and the Primer-BLAST program^2^. The S6F (12655–12,674) and S6R (13011–12,992) primer sequences matched the sequence of the serogroup 6 gene (accession number: NZ_AOGP0100049.1) All PCRs were carried out with each primer (20 pmol), DNA template (50 ng/μL), 5 μL 5 × FIREPol®Master Mix (Solis Biodyne, Estonia), and 1 μL ultra pure water (negative control). Positive controls were used in each PCR series (Table 1). PCR products were shown under UV illumination using ethidium bromide. A 50 bp or 1 kb DNA ladder (SM0373, Thermo Scientific) was used to compare DNA sizes.

**Table 1:** Primer sequences, number of cycles, PCR conditions used in this study.

Target gene	Primer Sequence (5′- 3′)	Number of cycles PCR product	PCR product (bp)	References
16S rRNA	TGGGCAATACGAACTACTCGGG CTTTAATCGTATTCGCAG	40(95°C 30 sn, 54°C 30 s, 72°C 30 s) 72°C 10 min	227	(10)
Serotype 1	CATTTCCTTAGGTTCAGC CAAGTCATCGTAATGCCT		306	
Serotype 2	GGCATATCCTAAAGCCGT AGAATCCACTATTGGGCACC	95°C 15 min, 35 (94°C 30 s, 55°C 45 s, 72°C 1 min) 72°C 10 min	160	(13)
Serotype 6	TGAGAATTTCGACAGCACT ACCTTGGCATATCGTACC		78	
Serotype 6	CGAACGGGAAAACCCCAAAC GCGTGAGCCTGAATAAAGCG	95°C 15 min, 35 (94°C 1 min, 60°C 1 min, 72°C 1 min) 72°C 15 min	357	This study
Tetracycline (tetH)	ATACTGCTGATCACCGT TCCCAATAAGCGACGCT	95°C 5 min; 30 (94°C 30 s, 60°C 1 min, 72°C 1 min) 72°C 8 min	1,086	(16)
Ampicillin (blaROB-1)	AATAACCCTTGCCCCAATTC TCGCTTATCAGGTGTGCTTG	95°C 5 min; 30 (94°C 30 s, 60°C 1 min, 72°C 1 min) 72°C 8 min	675	(16)
A quinolone (gyrA)	TTCAATGAGCGAATTAGCCA TCAGGAATCATCTCTTTGCC	94°C 5 min 35 (94°C 30 s, 55°C 1 min, 72°C 1 min) 72°C’de min	474	(17)
A quinolone (parC)	GATGGCTTGAAACCGGTGCA GCCATTCCCACCGCAATCC	94°C 5 min 35 (94°C 30 s, 55°C 1 min, 72°C 1 min) 72°C’de min	425	(17)
Tilmicosin (ermX)	GAGATCGGRCCAGGAAGC GTGTGCACCATCGCCTGA	95°C 5 min; 30 (94°C 30 s, 58°C 1 min, 72°C 1 min) 72°C 8 min	488	(18)
Neomycin (aphA-1)	TTATGCCTCTTCCGACCATC GAGAAAACTCACCGAGGCAG	95°C 5 min; 30 (94°C 30 s, 54°C 1 min, 72°C 1 min) 72°C 8 min	489	(19)
Leukotoxin	GTCCCTGTGTTTTCATTATAAG CACTCGATAATTATTCTAAATTAG	95°C 5 min, 40 (95°C 1 min, 53°C 1 min, 72°C 1 min) 72°C 15 min	385	(14)
ORF*lkt*	ATGGGTAATAAACTTACTAATATTTC TCATTAAGCTGCTCTAGCAAATTG	94°C 15 min, 40 (94°C 60 s, 55°C 1 min, 72°C 2 min) 72°C 15 min	3,000	This study
Serotype 1-specific antigen	TTCACATCTTCATCCTC TTTTCATCCTCTTCGTC’ 3	94°C 15 min 40 (95°C 1 min, 48°C 1 min, 72°C 2 min) 72°C 15 min	327	(15)
ORF*ssa-1*	ATGTATAAAATAAAGCATTC TCATTAGAAACTAAAGCCAACATTTAC	94°C min, 40 (94°C 1 min, 51°C 1 min, 72°C 2 min), 72°C 15 min	3,000	This study
SUMO Forward T7 Reverse	AGATTCTTGTACGACGGTATTAG TAGTTATTGCTCAGCGGTGG	94°C min, 40 (94°C 1 min, 60°C 1 min, 72°C 2 min), 72°C 15 min	3,250	Invitrogen, USA

### Sequence analysis of 16S rDNA, serogroup, *lkt*, and *ssa-1* genes

Sequence analysis of 16S rDNA, serogroup, *lkt*, and *ssa-1* genes was performed with primers for genotyping and the selection of candidate vaccine strains. Sequencing was performed at another laboratory (BM Metabion, Turkey). The similarity of each sequence of the 16S rDNA, *lkt*, and *ssa-1* genes was evaluated by accession numbers (NR_114448.1, AF314516.1, and U07788.1, respectively) in NCBI. The results were compared using LALIGN EMBL-EBI and Clustal 2.1 multiple sequence alignment programs. In addition, the recombinant plasmids were verified by PCR and sequence analysis using ORF*lkt*, ORF*ssa-1*, SUMO forward, and T7 reverse primers (Table 1).

### Selection criteria for S1, S2 and S6 *Mannheimia haemolytica* master vaccine seeds

To increase the effectiveness of the candidate vaccine, criteria such as serotype, colony morphology, hemolytic activity, slime production, resistance to antimicrobial agents, the difference in *lkt*, and *ssa-1* virulence proteins, the similarity of 16S rDNA were significant in the determination of master seed strains for candidate vaccines in this study. Similarly, recombinant *lkt* and ssa-1 proteins can be universal. Accession numbers (NR_114448.1, AF314516.1, U07788.1) were used to evaluate the sequence results of the 16S rDNA, *lkt*, and *ssa-1* genes, respectively.

Clustal 2.1 Multiple Sequence Alignment program was used to compare the nucleotide sequences of the strains with sequences of reference genes in NCBI. Amino acid sequences were evaluated with reference strains using UniProt Knowledgebase (UniProtKB)/Swiss-Prot SIB Swiss Institute of Bioinformatics. The target strains and genes were determined based on the results obtained for the preparation of these vaccines.

### Production of *lkt* and SSA-1 recombinant proteins

The ORF*ssa-1* and ORF*lkt* primers (Table 1) were designed with accession numbers AF314516.1 and U07788.1, respectively, in NCBI. The PCR product was then purified using a GenElute Gel Extraction Kit (Sigma-Aldrich, Germany), and the pure PCR product was cloned using a Champion pET SUMO Protein Expression System (Invitrogen, USA). Plasmids from randomly selected recombinant colonies on agar were isolated using the GenElute Plasmid Miniprep Kit (Sigma-Aldrich, Germany) according to the manufacturer’s protocol. The recombinant plasmid was verified by PCR and sequence analysis using ORF*ssa-1* and ORF*lkt*, SUMO forward, and T7 reverse primers (Table 1). Sequencing was performed at another laboratory (BM Metabion, Turkey). The recombinant *lkt* (r*lkt*) and SSA-1 (rSSA-1) proteins were produced as previously described (20). Briefly, the first culture obtained by incubating the confirmed recombinant colony in LB medium containing 50 μg/mL kanamycin for 16 h at 37°C, was incubated in TY medium containing 50 μg/mL kanamycin (1:9, first culture: TY) (Tryptone. 5.0 g, yeast extract. 3.0 g, CaCl2 x 2 H2O. 0.9 g, distilled water 1 L. pH 6.8.) at 37°C and 200 rpm until the optical density (OD) 550 = 0.6–0.8. Then, IPTG was added to the medium to a final concentration of 0.1 mM (AppliChem, Germany) and incubated in a water bath for 4 h at 30°C and 200 rpm. The control culture was processed using the same protocol without IPTG.

To determine whether the protein was soluble or insoluble, protein profiles were examined using sodium dodecyl sulfate-polyacrylamide gel electrophoresis (SDS-PAGE) and Western blotting.

### Preparation of seven candidate vaccines and application on mice groups

In order to define the best culture media to produce the vaccine strains, the differences in the amount and variety of proteins produced by each of the S1, S2, and S6 serotypes in BHI (110,493, Merck), 2TY (bacto tryptone 16 g., bacto yeast extract 10 g., NaCl 5 g., 1 L distilled H_2_O, pH 7.0), and Todd Hewitt broth media (CM0189B, Oxoid) were evaluated. Candidate vaccines were prepared using logarithmic-phase culture supernatant fluids from the *M. haemolytica* serotype as described previously (9, 21). The protein profiles obtained from cultures were analyzed by SDS-PAGE (22). To compare protein molecular weight, PageRuler™ Plus Prestained Protein Ladder, 10 to 250 kDa (26,619, Thermo Scientific) was used.

In this study, seven candidate vaccines were developed as monovalent (*n* = 3), bivalent (*n* = 1), trivalent (*n* = 1), and recombinant (*n* = 2) vaccine types (Table 2). Culture supernatant proteins were concentrated using solid ammonium sulfate (40%), and pellet antigens of three serotypes were obtained using a previously described procedure (23–25). The protein concentrations were determined using a BCA protein assay kit (A55860, Thermo Scientific) according to a previously described procedure (26).

**Table 2:** Components of the seven candidate vaccines developed in this study, as well as the contents of the two commercial vaccines used for comparison of efficiencies.

Vaccine type	Vaccine name↓	Strain type	Bacterin CFU/mL	Concentrated supernatant protein (μg/mL) in one dose	Recombinant protein (μg/mL)	Adjuvant type	Formulation Antijen solution/adjuvant (v/v)
Monovalent	S1	Serotype 1	1 × 10^10^	20 μg/mL	–	ISA-206*	50/50
	S2	Serotype 2	1 × 10^10^	20 μg/mL	–	ISA-206*	50/50
	S6	Serotype 6	1 × 10^10^	20 μg/mL	–	ISA-206*	50/50
Recombinant	ISA206	–	–	–	*ssa-1* (14 μg/mL) + *lkt* (70 μg/mL)	ISA-206*	50/50
	FCA	–	–	–	*ssa-1* (14 μg/mL) + *lkt* (70 μg/mL)	FCA**	50/50
Bivalent	B	Serotype 1	1 × 10^10^	20 μg/mL	–	ISA-206*	50/50
		Serotype 2	1 × 10^10^	20 μg/mL			
Trivalent	T	Serotype 1	1 × 10^10^	20 μg/mL			50/50
		Serotype 2	1 × 10^10^	20 μg/mL	*ssa-1* (14 μg/mL) + *lkt* (70 μg/mL)	ISA-206*	50/50
		Serotype 6	1 × 10^10^	20 μg/mL			50/50
Commercial	C1	Serotype 1	1 × 10^9^				
		Serotype 2	1 × 10^9^	Unknown	–	AlOH***	Unknown
		*Pasteurella multocida*	1 × 10^9^				
	C2	Serotype 1	1 × 10^9^				
		BRS virus	TCID50 = 10^5.5^	Unknown	–	AlOH	Unknown
		Parainfluenza 3-Virus	TCID50 = 10^7.3^				

* Montanide TM ISA 206 VG. ** Freund’s Complete Adjuvant. *** Aluminium hydroxide gel.

Montanide™ ISA 206 VG (Seppic, Castres, France) (ISA 206) adjuvant and Freund’s complete adjuvant (F5881, Sigma-Aldrich, Germany) (FCA) were used to prepare those vaccines (Table 2).

#### Sterility test

Aerobic, anaerobic, microaerophilic bacteria, *Mycoplasma* spp., and fungi were cultured in appropriate liquid media (25 mL broths in 50–100 mL Erlenmeyer flasks) and solid media and incubated at appropriate incubation temperatures for 14 days. No growth was observed, indicating the sterility of the candidate vaccine (23, 24).

#### Safety test

Candidate vaccines (56 mice in total, 8 mice in each vaccine group); sterile Montanide™ ISA 206 VG (control Group 1) (8 mice); sterile phosphate buffer saline sterile PBS (control Group 2) (8 mice) were injected intraperitoneally, and the mice were observed for 7 days. All experimental animals were observed for 7 days and followed up for unusual clinical findings (sudden death, restriction of movements, loss of appetite, depressive reactions, frequent urination, etc.). The absence of clinical findings in any of the animals was the harmlessness test criterion of the vaccine (27).

Female Swiss albino mice weighing 15–18 g were used in this study. The sample size was calculated with the G-Power package program (3.1.9.7) with an effect size of 0.9, an error of 0.05, and a power of 85%, 30 mice per group. The efficiency of each candidate vaccine was investigated in the challenge (*n* = 18), seropotency (*n* = 6), and control (*n* = 6) mouse groups (28, 29). Mice were vaccinated intramuscularly with each adjuvanted 0.2 mL of vaccines twice at 21-day intervals. An equivalent amount of physiological saline was injected into the control group. The 50% lethal dose (LD_50_) values for each S1, S2, and S6 strain were determined as previously described (9, 26, 27). To determine cross-protection, challenge groups of each candidate vaccine were tested with these three strains. These serotypes were used for the challenge experiments in each vaccine group.

In addition, the protective efficacy and immunogenicity of the seven candidate vaccines were compared with the results of two commercial vaccines (Table 2).

### Analysis of vaccinated mice groups

In-house-made ELISA was developed to investigate anti-*M. haemolytica* IgG levels in the vaccinated mice sera. To compare the effectiveness of antigen differences on serological diagnosis, eight different antigens (whole pellet antigens and supernatant proteins of each one of S1, S2, and S6 serotype and recombinant *lkt* (r*lkt*) and rSSA-1) were used. According to antibody levels, the best coating ELISA antigen was determined. This application aimed to determine the most sensitive antigen for assessing antibody levels induced by vaccines. The highest dilution of serum yielding an OD450 value equal to or greater than twice that of a comparable negative control serum dilution was considered the serum’s antibody titer. The antigen amount and sera dilution rate with conjugates were standardized by the checkerboard titration method (25–30).

After giving live bacteria in the LD50 value to the challenge groups, the internal tissues of these groups were collected in 20 mL of sterile saline solution on the tenth day, and we calculated then the bacterial clearance power of each candidate vaccine from these tissues (26, 28).

Vaccine efficacy is measured by calculating the incidence rates of disease among vaccinated and unvaccinated mice and determining the percentage reduction in the incidence rate of disease among vaccinated mice compared to unvaccinated mice (29, 30). The seropotency vaccine efficacy (SVE) and challenge vaccine efficacy (CVE) were measured using a basic formula (31):


$$\begin{array}{l} \mathrm{Seropotency vaccine efficacy}\;\left(SVE\right)\\ =\frac{\mathrm{number of serum above the}\;\mathrm{cut}-\mathrm{off}\;\mathrm{value}}{\mathrm{total mice in}\;a\;\mathrm{vaccine type}}\times 100 \end{array}$$

$$\mathrm{Challenge vaccine efficacy}\;\left(CVE\right)=\frac{DRU–DRV}{DRU}\times 100$$

Death rate in the unvaccinated population = DRU.

Death rate in the vaccinated population = DRV.

### Statistical analysis

Cut-off values of each coating antigen of the in-house ELISA were calculated using receiver operating characteristic (ROC) analysis (95% confidence intervals (CI) and *p*-value of 0.05). Then, to determine the most suitable coating antigen and the antibody stimulation of vaccine models, one-way analysis of variance (ANOVA) was used based on Levene’s homogeneity test after the differences between the vaccine groups were evaluated by *post hoc* Tamhane’s T2 test (*p* < 0.001). The relative risk value contains the odds ratio of vaccine efficacy, which is the most important determinant in detecting the most effective vaccines (together with seropotency, challenge, and control groups of each vaccine). Therefore, multinomial logistic regression was used to determine the RR of each candidate vaccine (13, 14, 16, 32).

## Results

### Phenotypic and genotypic properties of the strains isolated from sheep and cattle

A total of 65 (13%) *M. haemolytica* strains were identified by biochemical tests and 16S rDNA analysis using PCR. These strains were isolated from calves (44.61%) and sheep (55.38%) lung samples. The macroscopic morphology obtained from these isolates was 67–69% smooth (n = 44) and 32–31% mucoid (n = 21). Six (9.23%) isolates, which were classified as S6, synthesized biofilms. Although hemolytic activity was detected in 46.15% of colonies, it was not related to the serotype of the strain or the presence of the *lkt* gene. Hemolytic activity was detected in 30 (46.15%) strains, but the *lkt* gene was detected in 16 (33.84%) of these strains.

As a result of the antibiotic resistance of strains, only quinolone resistance genes, gryA with 58.46% (*n* = 38), and parC with 47.69% (*n* = 31) containing colonies, were determined using PCR. According to the disc diffusion test, the strains were detected against antimicrobial agents such as amoxicillin-clavulanic acid (S = 90.77%, R = 9.23%), ceftriaxone (S = 84.61%, R = 15.39%), gentamicin (S = 15.38%, R = 84.61%), cefotaxime (S = 87.7%, R = 123%), ceftazidime (S = 58.46%, R = 41.53%), ampicillin (S = 52.31%, R = 47.69%), and ciprofloxacin (S = 55.39%, R = 44.61%). When the development of resistance against at least one or two antimicrobial groups in the isolates was evaluated, 32 of them were MDR (49.23%), and 29 were XDR (44.61%), while 4 of them (6.15%) were sensitive to all antimicrobial agents (Table 3).

**Table 3:** Investigation of the phenotypic (serotype, colony morphology, haemolytic activity, slime production, resistance to antimicrobial agents) properties and virulence genes in *Mannheimia haemolytica* strains.

No	Sample number	Colony type	Serotype	Hemolysis activity	Biofilm	Leukotoxin gene	S1- specific Antigen gene	Antibiotic-resistant*
1	M1	Mukoid	6	+++	−	−	+	MDR
2	M3	Mukoid	2	−	−	+	+	XDR
3	M8	Mukoid	6	+	+	+	+	MDR
4	M11	Mukoid	6	−	−	+	+	MDR
5	M12	Mukoid	?	−	−	−	+	XDR
6	M35	Smooth	6	−	−	−	+	−
7	M51	Smooth	6	++	−	−	+	MDR
8	M57	Smooth	6	−	−	−	−	−
9	M60	Smooth	6	−	−	−	−	XDR
10	M64	Smooth	6	−	−	−	+	MDR
11	M66	Smooth	6	−	−	−	+	MDR
12	M72	Smooth	6	−	−	−	−	XDR
13	M87	Smooth	6	++	+	−	−	MDR
14	M109	Smooth	2	+	−	−	−	MDR
15	M110	Smooth	1	++	−	+	+	XDR
16	M111	Smooth	?	++	−	−	−	MDR
17	M112	Smooth	2	++	−	+	+	MDR
18	M113	Smooth	2	++	−	+	−	XDR
19	M114	Smooth	2	++	−	+	−	MDR
20	M115	Smooth	2	++	−	+	−	XDR
21	M116	Smooth	2	++	−	+	+	MDR
22	M117	Smooth	2	++	−	+	+	MDR
23	M118	Mukoid	2	−	−	+	+	XDR
24	M119	Smooth	6	−	+	−	−	MDR
25	M120	Mukoid	2	−	−	−	−	MDR
26	M121	Smooth	1	−	−	−	−	XDR
27	M122	Smooth	6	−	−	−	−	XDR
28	M123	Mukoid	2	−	−	−	−	XDR
29	M128	Mukoid	2	++	−	−	−	XDR
30	M129	Mukoid	1	++	−	−	−	XDR
31	M130	Smooth	1	−	−	−	−	XDR
32	M131	Mukoid	2	−	−	+	+	XDR
33	M132	Mukoid	6	+++	−	−	+	MDR
34	M133	Smooth	6	−	−	−	+	−
35	M134	Mukoid	6	+	−	+	+	MDR
36	M135	Mukoid	6	−	−	+	+	MDR
37	M136	Mukoid	?	−	−	−	+	XDR
38	M137	Mukoid	2	−	−	+	+	MDR
39	M138	Smooth	6	++	−	−	+	MDR
40	M139	Smooth	6	−	+	−	−	−
41	M140	Smooth	6	−	−	−	−	XDR
42	M141	Smooth	6	−	−	−	+	XDR
43	M142	Smooth	6	−	−	−	+	MDR
44	M143	Smooth	6	−	−	−	−	XDR
45	M144	Smooth	6	++	+	−	−	MDR
46	M145	Smooth	2	+	−	−	−	MDR
47	M146	Smooth	1	++	−	+	+	XDR
48	M147	Smooth	?	++	−	−	−	MDR
49	M148	Smooth	2	++	−	+	+	MDR
50	M149	Smooth	2	++	−	+	−	XDR
51	M150	Smooth	2	+++	−	+	−	MDR
52	M151	Smooth	2	+++	−	+	−	XDR
53	M152	Smooth	2	++	−	+	+	MDR
54	M153	Smooth	2	++	−	+	+	MDR
55	M154	Smooth	6	−	+	−	−	MDR
56	M155	Mukoid	2	−	−	−	−	MDR
57	M156	Smooth	1	−	−	−	−	XDR
58	M157	Smooth	6	−	−	−	−	MDR
59	M158	Mukoid	2	−	−	−	−	MDR
60	M159	Mukoid	2	++	−	−	−	XDR
61	M160	Mukoid	1	++	−	−	−	XDR
62	M161	Smooth	1	−	−	−	−	XDR
63	M162	Smooth	?	−	−	−	−	XDR
64	M163	Mukoid	6	−	−	−	−	XDR
65	M164	Smooth	1	−	−	−	−	XDR
66	ATCC 43270	Smooth	1	−	−	−	+	XDR
67	ATCC 29694	Smooth	2	−	−	−	−	XDR
68	ATCC 29697	Smooth	6	−	−	−	−	−

*Extensively-drug resistance (XDR); Multi-drug resistance (MDR).

When the serotype of the strains was compared with band sizes as per previously described methods (13), the size of each PCR fragment corresponded to only *M. haemolytica* ATCC 43270 S1 (306 bp) and *M. haemolytica* ATCC 29694 S2 (160 bp). The serotype of 32 isolates (50.76%) was defined using a previously described method (13). The S1 serotype (10.76%) was detected in both the sheep (*n* = 4) and calf (*n* = 3) samples. The S2 serotype (38.46%) was isolated from sheep (*n* = 20) and calf (*n* = 5) samples. However, we could not identify the S6 serotype previously described methods (13). By the new-designed PCR protocol in this study, 28 non-typical strains were identified as S6 serotypes (43.07%), and these were found in sheep (*n* = 9) and calf (*n* = 19) samples.

After 2016, an increase in the percentage of S6 serotypes was detected. Five isolates (7.69%) were not S1, S2, or S6 and were therefore considered to belong to other serovars or were not typable using both PCR protocols. *Lkt* and SSA-1 genes were detected, respectively, in 22 (33.84%) and 28 (43.07%) of the strains.

### Evaluation and selection of master seed stocks

The selection of *M. haemolytica* master seeds was a multi-stage process. The goal was to determine the 3 different serotypes that show diverse phenotypic and genotypic characteristics. As a result of the selection of *M. haemolytica* master seeds, the S1 (M110), S2 (M123), and S6 (M8) strains were determined (Table 3).

**Figure 2:**
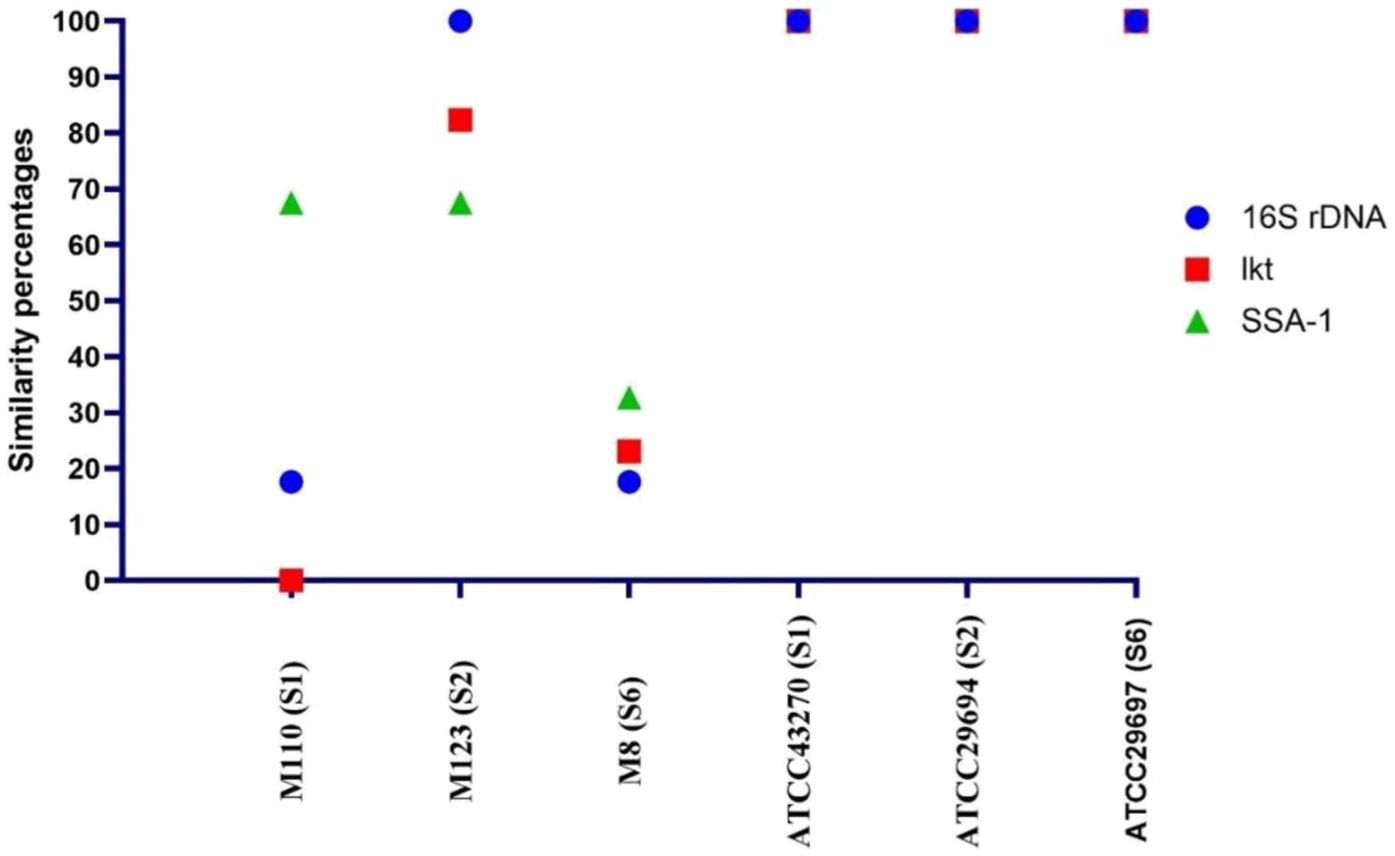
Similarity between the reference strains and the master seed strains according to the sequence analysis results.

According to the sequence results, it was determined that the highest similarity with the reference strains was presented by the M123 (S2) strain, while the M8 (S6) strain was different from the reference strains and other vaccine strains. In addition, the genes of the M123 (S2) strain were used to produce *lkt* and SSA-1 recombinant proteins because they were very similar to the reference strain. In contrast, it was thought that the M8 (S6) strain would be useful to use in vaccines due to the sequence difference, therefore it was included in the vaccine formulation (Figure 2).

**Figure 3:**
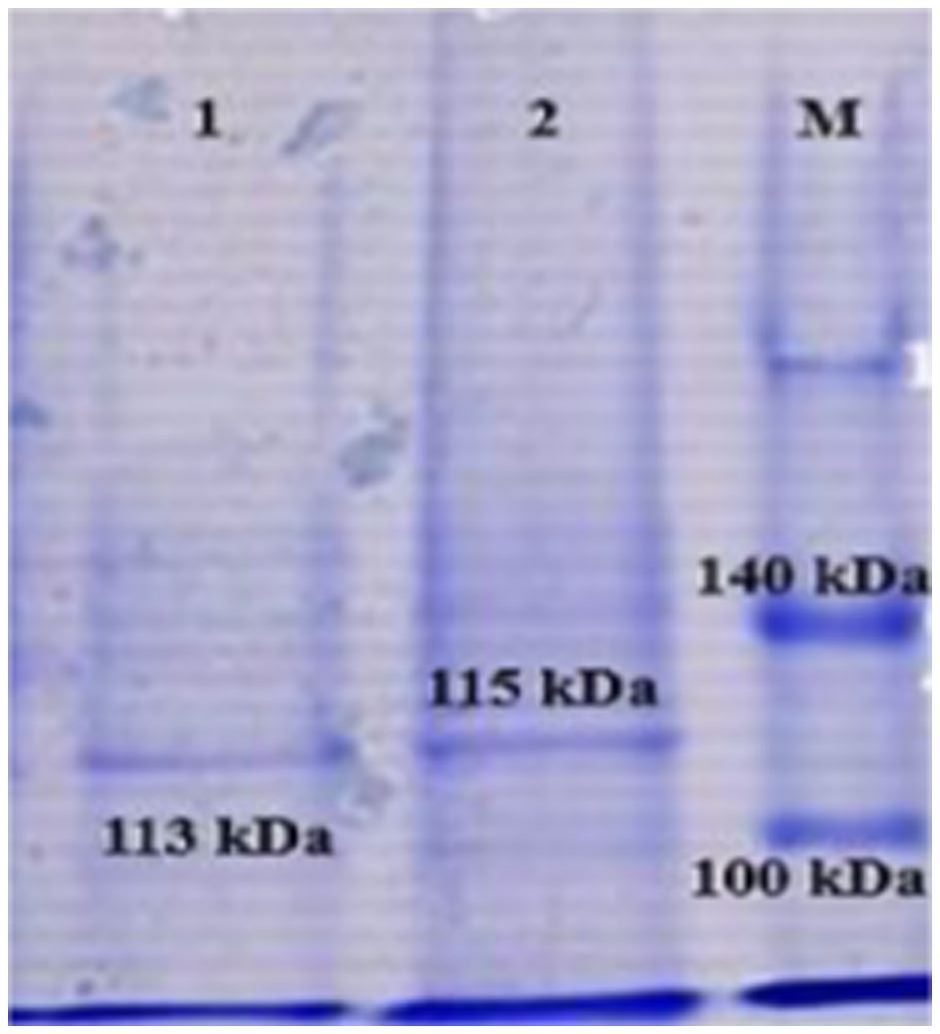
Demonstration of recombinant leukotoxin (*lkt*) and S1-specific antigen (SSA-1) proteins by SDS-PAGE.

The recombinant proteins obtained by SDS-PAGE were determined to be in soluble form. When the molecular weights of the recombinant proteins were evaluated, the presence of pure recombinant protein was detected with a weight of 113 kDa for rlkt and 115 kDa for rssa-1, together with the SUMO protein (11 kDa) (Figure 3).

**Figure 4:**
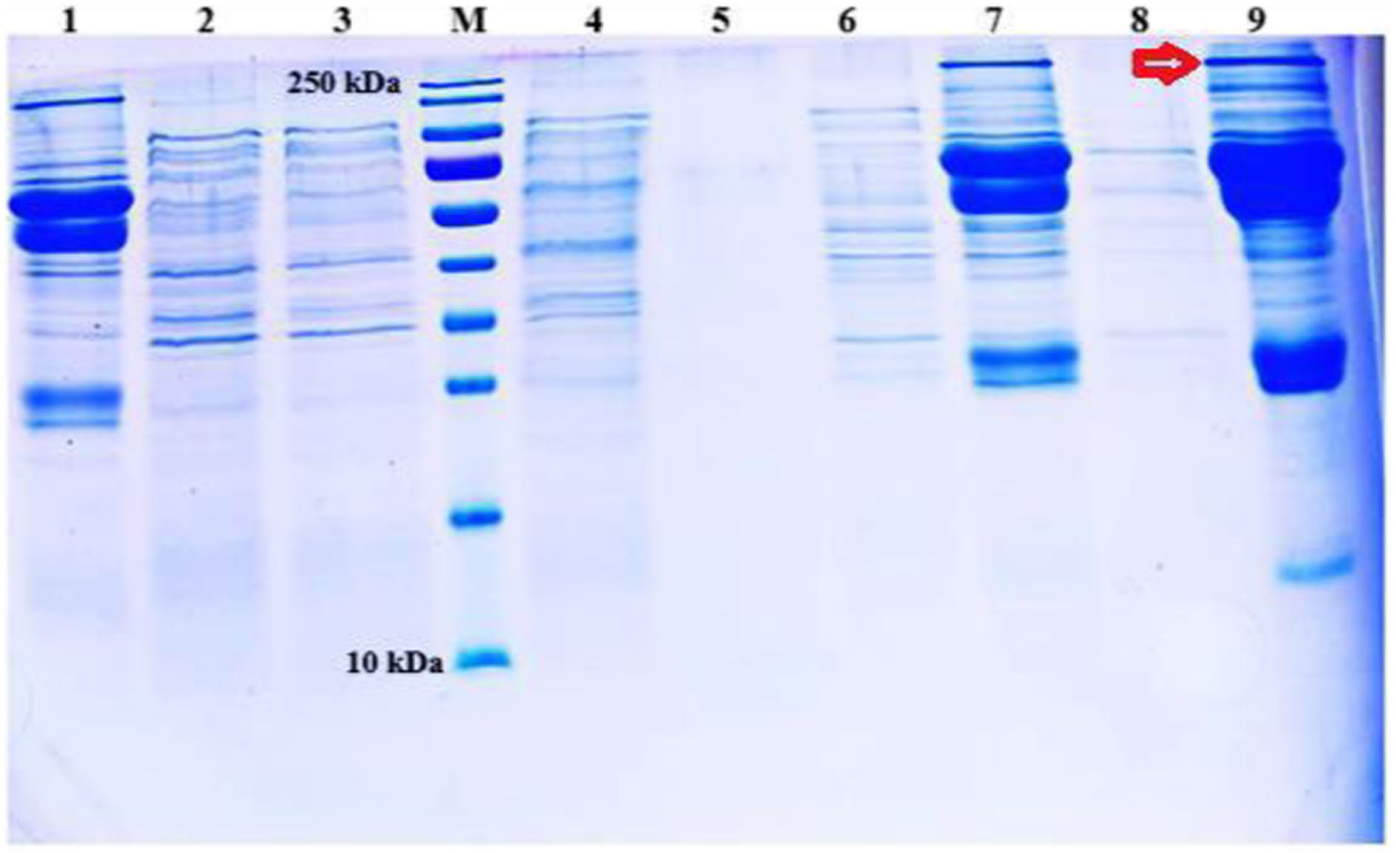
Analysis of protein profiles of S1, S2, and S6 *M. haemolytica* vaccine strains produced in three different media and determination of the ideal media. Protein weighing approximately 250 kDa is indicated by the arrow. 1: S1 strain in Todd Hewit’s broth, 2: S1 strain in BHI, 3: S1 strain in 2TY, 4: S2 strain in Todd Hewit’s broth, 5: S2 strain in 2TY, 6: S2 strain in BHI, 7: S6 strain in Todd Hewit’s broth, 8: S6 strain in 2TY, 9: S6 strain in BHI.

According to SDS-PAGE, host specificity protein J, weighing 250 kDa, was, interestingly, detected in the S6 serotype incubated in Todd Hewit’s broth and BHI. Since this protein caused widespread bleeding in the internal organs of experimental mouse groups, adding this toxin to the candidate vaccine was considered. Additionally, when the protein synthesis induction for each serotype was evaluated, it was decided to produce all the master seed strains in Todd Hewit’s broth as per previously described protocols (9) (Figure 4).

### Results of seropotency and challenge groups

**Figure 5:**
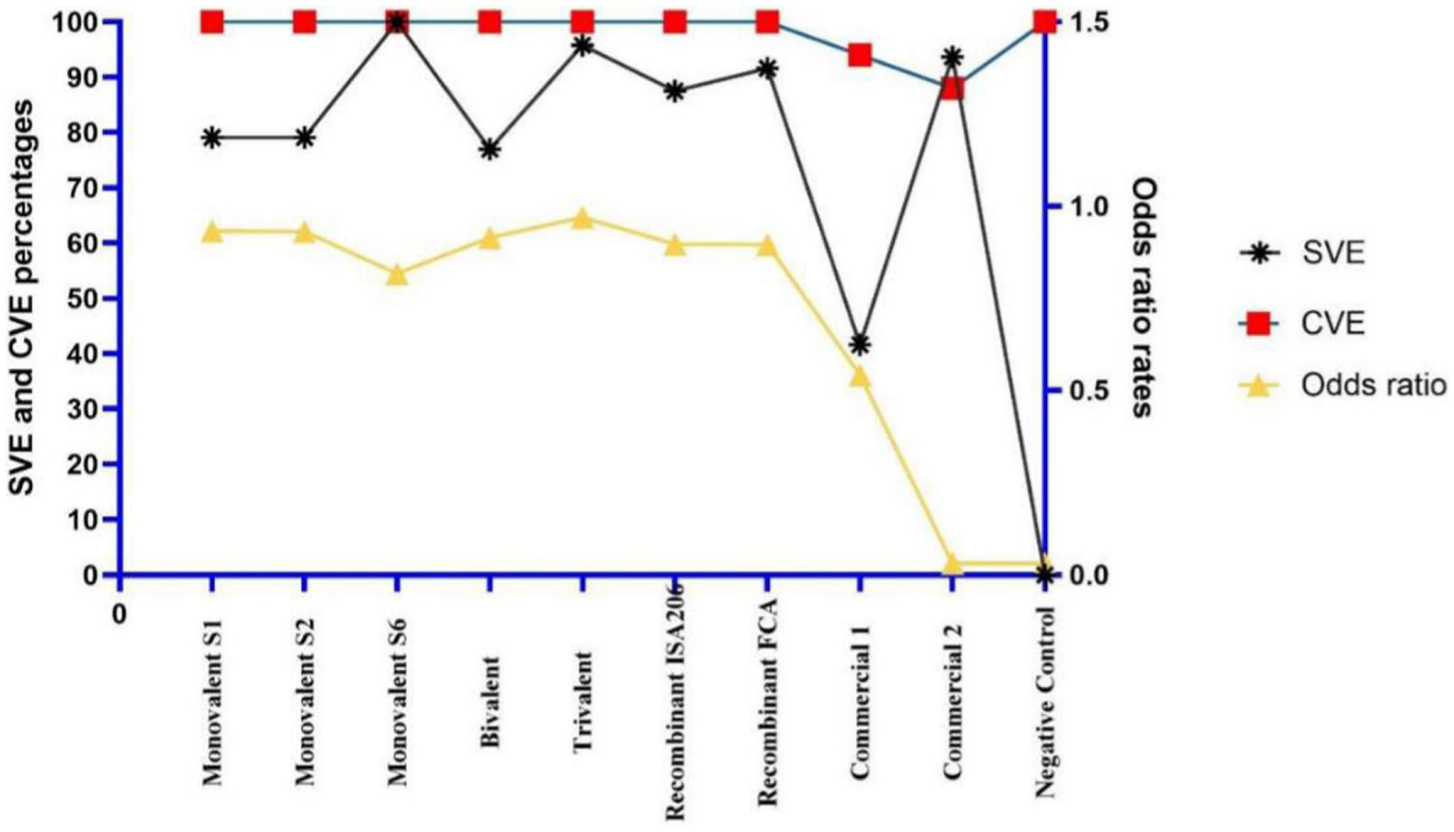
Comparison of candidate vaccines according to seropotency vaccine efficiencies (SVE), challenge vaccine efficiencies (CVE), and odds ratios.

ELISA was performed to detect the best coating antigen, cut-off value, and effectiveness of the vaccine groups. With the discriminatory power of eight coating antigens, the S2 pellet antigen displayed the highest OD value in the analysis of sera of potency groups. The cut-off value of S2 pellet antigen-based ELISA was 114.5 according to ROC curve analysis values on AUC 1. In addition, a positive likelihood of 41, a sensitivity of 100%, a specificity of 98%, a standard error of 0, and a confidence level of 100% were observed (Table 4). Compared with the seven vaccine groups, a significant difference (*p* value <0.05) was determined between groups. The highest SVE, 100%, was obtained from both S6 monovalent and trivalent vaccines (Table 5). Interestingly, in the S6 strain challenge groups, only both groups of commercial vaccines resulted in mortality. Considering the SVE, CVE, and odds ratio values, all the candidate vaccines were better than commercial vaccines, the trivalent vaccine type being the most effective vaccine type (Table 4 and Figures 4, 5).

**Table 4:** Determination of anti-*Mannheimia haemolytica* IgG levels in vaccinated mouse sera using the in-house ELISA developed with eight different coating antigens.

Type of vaccine →		Monovalent			Multivalent		Recombinant		Commercial		Negative control
Subtype of vaccine →		S1	S2	S6	Bivalent	Trivalent	ISA 206	FCA	C1***	C2	
Test antigen ↓	Cut-off value ↓	Mean.*± SD**	Mean.*± SD**	Mean.*± SD**	Mean.*± SD**	Mean.*± SD**	Mean.*± SD**	Mean.* ± SD**	Mean.*± SD**	Mean.*± SD**	
S1 (Pellet)	201.5	318.3 ± 65.3	285.1 ± 74.7	468.6 ± 31.5	217.8 ± 52.4	397 ± 72.8	197.1 ± 44.4	197.5 ± 54	551 ± 57.9	612.3 ± 47.1	114 ± 18.7
S2 (Pellet)	114.5.	390.6 ± 35.6	380.1 ± 100.3	590.6 ± 36	292.6 ± 76.9	548.5 ± 34	226.8 ± 57.3	225.1 ± 35.5	563.6 ± 97.7	648.3 ± 35	108.2 ± 20.1
S6 (Pellet)	137.5	129.8 ± 18	137 ± 29.8	540 ± 54.5	101.6 ± 7.7	181.1 ± 46.8	134 ± 13.6	139.3 ± 33.8	62.1 ± 10.3	140.8 ± 25.9	113.4 ± 10.5
S1 (Supernatant)	201.5	264.6 ± 46.9	278 ± 40.6	255.6 ± 81	295.8 ± 120.7	247 ± 43.8	126.6 ± 11.2	135 ± 12.8	85.3 ± 5.2	176.3 ± 24.1	112.7 ± 20.5
S2 (Supernatant)	159.5	121.1 ± 23.7	131 ± 25	157.8 ± 44.5	143.1 ± 47.8	164 ± 57.4	144 ± 21.9	151 ± 26.5	67 ± 14.6	124.6 ± 49.4	124.8 ± 5.2
S6 (Supernatant)	121.5	84 ± 10.5	98.5 ± 3.6	201.8 ± 59.5	127.8 ± 27.1	511.3 ± 42	118.6 ± 19	120.1 ± 11.4	100.6 ± 13.7	194.3 ± 27.3	119 ± 5.2
Rssa	141	147.5 ± 27	159.6 ± 13.3	209 ± 41.3	152.5 ± 33.4	183.8 ± 21.4	151 ± 41.3	181.1 ± 47.6	140.5 ± 49.2	189 ± 18	114.6 ± 10.5
R*lkt*	213.5	248.6667 ± 56.9	298.8 ± 42.8	302.3 ± 68.7	251.5 ± 55.8	236 ± 52.7	200.8 ± 72.2	271.5 ± 101.4	213.5 ± 44.7	360.3 ± 73.4	106 ± 20.2

*Arithmetic mean. **Standard deviation. *** Commercial vaccine (C1) results were used as positive control.

**Table 5:** Determination of seropositive vaccine efficacies, vaccine challenge efficacies, and odds ratios of vaccines studied.

		Monovalent			Multivalent		Recombinant		Commercial		Negative control
Type of vaccine →		S1	S2	S6	Bivalent	Trivalent	ISA206	FCA	C1	C2	
SVE ^*^		79.1% (40/48)	79.1%(40/48)	100%(48/48)	77%(40/48)	95.8%(48/48)	87.5%(45/48)	91.6%(45/48)	41.6%(20/48)	93.7%(45/48)	0%
CVE**	Challenge groups	100%	100%	100%	100%	100%	100%	100%	94%	88%	100%
Odds ratio***		0.933	0.931	0.817	0.915	0.970	0.897	0.896	0.542	0.032	0.0032

* *Seropotency vaccine efficacy = For each vaccine type six mice were used*. $SVE=\frac{\mathrm{number of sera above the}\;\mathrm{cut}-\mathrm{off}\;\mathrm{value}}{\mathrm{total mice in}\;a\;\mathrm{vaccine}-\mathrm{type}}\times 100$ . ** Challenge vaccine efficacy = For each vaccine type, it was performed using a total of 18 mice [serotype 1 (*n* = 6), S2 (*n* = 6), S6 (*n* = 6)], and control group (*n* = 6). $CVE=\frac{\mathrm{death rate in the unvaccinated population}\;\left(DRU\right)-\mathrm{death rate in the vaccinated population}\;\left(DRV\right)}{DRU}\times 100$ . *** Multinomial logistic regression was used for the determination of the RR (odds ratio) of each candidate vaccine.

## Discussion

*Mannheimia haemolytica* is one of the most important pathogens of cattle and sheep’s respiratory system. To control and prevent the disease caused by *M. haemolytica*, the development of broad-spectrum diagnostic tools and effective vaccines is essential. In this study, we identified phenotypic and genotypic diversity in field isolates of *M. haemolytica* and determined master seed strains for vaccine development based on the properties of these strains.

When comparing serotype diversity in animal hosts with Mannheimia-associated pneumonia, this diversity is greater in small ruminants. *M. haemolytica* S1, S2 and S6 are the most prevalent worldwide (13). However, S6 has emerged as an important serotype more recently in both cattle and sheep (6, 33). In this study, the highest percentage of *M. haemolytica* S6 strains was determined in cattle higher than in sheep, contrary to previous studies (7, 11, 34, 35). In addition, the S2 serotype obtained from sheep samples was detected at a higher percentage than in calves, and this result was similar a previous report (13). Further, the increase in the S6 rate could be related to a genetic change in the ability of the serotype to colonize and proliferate in the upper respiratory tract and subsequently induce lung lesions (6, 36). Thus, the development of broad-spectrum vaccines containing predominant and emergent serotypes is essential for the effective control of *M. haemolytica* infections in cattle and small ruminants.

*Mannheimia haemolytica* commercial vaccines, primarily prepared with S1 and/ or S2 serotypes and containing different amounts of *lkt*, are moderately effective in protecting against this infection (37–39). *M. haemolytica* S1 vaccines appeared efficacious in approximately 50% of the field studies (38). *M. haemolytica* S2 strains demonstrated poor immunogenicity (9, 40). In addition, cross-serotype protection is not usually offered by *M. haemolytica* vaccines (9, 38, 41). S1 and S6 isolates indicate that they may be potential targets for serotype-specific identification and vaccine development (38). Because the serotype of commercial vaccine strains is not similar to the field stains serotypes, these vaccines cannot protect effectively against pneumonia caused by *M. haemolytica* (3, 9, 40). Increasing the efficacy of *M. haemolytica* commercial vaccines can be supported by recombinant *lkt*A protein (37) owing to the low yields of *lkt* in commercial vaccines (39, 41). However, insufficient *lkt* yield remains a significant engineering problem, and further bioprocess optimization is required to increase efficiency (42). Additionally, LPS may act synergistically with *lkt*, enhancing its effects and contributing to its endotoxic activity (43). Moreover, *lkt* diversity exists among the ovine strains of *M. haemolytica* (37). Although *β*-hemolysis is a reliable indicator of leukotoxicity, there are conflicting reports on which leukotoxin-deletion mutants can present hemolytic properties (6). Owing to an overall dissimilarity of 12% among *lkt* genes from several isolates (38–43), challenge trials in cattle have revealed that vaccination with r*lkt* alone failed to protect against the development of clinical symptoms (37). SSA-1 antigen, which likely has protease activity (7), is the most immunoreactive candidate among S1, S2, and S6 (33, 39). This protein colonizes the nasopharynx (9). Although it elicits a strong antibody response in both rabbits and cattle (7), it has also been shown to be downregulated 27-fold during *in vivo* infections (38). According to the literature, live-attenuated vaccines have shown partial efficacy against pasteurellosis, but killed vaccines can provide better protection (4, 9, 40, 44). An effective vaccine should stimulate both humoral and cell-mediated immune responses, providing sterilizing immunity and preventing disease (45). Vaccines containing multiple serotypes may provide broader protection than monovalent vaccines (40, 44). In this study, the locally developed polyvalent vaccine containing S1, S2 and S6 serotypes provided better protection against a lethal S6 challenge than the commercial vaccines that did not contain the S6 serotype.

Adjuvants, which are used as immunomodulators in vaccines, are classified into six main types based on their composition and mode of action (46). These include mineral salts, oil-in-water emulsions, water-in-oil emulsions, liposomes, microparticles, and cytokines (47). The choice of adjuvant can significantly impact the immune response elicited by the vaccine antigen.

In particular, a water-in-oil emulsion (W/O) can induce a strong and long-lasting humoral immune response (48). Adjuvants can be used in combination to further enhance the vaccine efficacy. Vaccines containing novel adjuvants have demonstrated improved efficacy against *Mannheimia haemolytica* compared to traditional aluminum hydroxide-based vaccines (40). W/O/W formulations, including Montanide™ ISA 206 VG, are continuous aqueous phase emulsions in which oil droplets contain a second aqueous phase (double emulsion) (49). This elicits a higher immune response. FMD vaccines adjuvanted with Montanide™ ISA 206 induce immüne response better than the vaccine adjuvanted with Quil-A Saponin (50). Polyvalent inactivated Pasteurella and Clostridial vaccine adjuvanted by Montanide™ ISA 206 VG provides protective immunity (51).

## Conclusion

This study demonstrated the superior protective efficacy of a novel trivalent *M. haemolytica* vaccine containing serotypes S1, S2, and S6, along with r*lkt*, and rSSA-1 proteins, adjuvanted with Montanide™ ISA 206, in a murine model. The inclusion of the S6 serotype, increasingly recognized for its prevalence and virulence, proved crucial for enhanced protection against a lethal S6 challenge, surpassing the efficacy of commercial vaccines lacking this serotype. This underscores the importance of incorporating prevalent and emerging serotypes in vaccine formulations for broader cross-protection, a significant challenge in *M. haemolytica* vaccine development. The enhanced efficacy observed with the trivalent vaccine may be attributed to the combination of key immunogenic proteins (r*lkt* and rSSA-1) and the potent immunostimulatory properties of Montanide™ ISA 206, which promotes both humoral and cellular immunity. These findings support the potential of this trivalent vaccine as a promising strategy for controlling *M. haemolytica* infections in sheep and cattle, particularly in settings where the S6 serotype is prevalent. Further research, including field trials, is required to evaluate the long-term efficacy and safety of this vaccine in target animal populations.

## Data Availability

The original contributions presented in the study are included in the article/supplementary material, further inquiries can be directed to the corresponding author/s.

## Footnotes

^1^ https://www.ncbi.nlm.nih.gov/tools/primer-blast

^2^ https://blast.ncbi.nlm.nih.gov/Blast
